# Disulfide-Linked Peptides for Blocking BTLA/HVEM Binding

**DOI:** 10.3390/ijms21020636

**Published:** 2020-01-18

**Authors:** Marta Spodzieja, Katarzyna Kuncewicz, Adam Sieradzan, Agnieszka Karczyńska, Justyna Iwaszkiewicz, Valérie Cesson, Katarzyna Węgrzyn, Igor Zhukov, Martyna Maszota-Zieleniak, Olivier Michielin, Daniel E. Speiser, Vincent Zoete, Laurent Derré, Sylwia Rodziewicz-Motowidło

**Affiliations:** 1 Faculty of Chemistry, University of Gdansk, Wita Stwosza 63, 80–308 Gdańsk, Poland; m.spodzieja@wp.pl (M.S.); k.kalejta@gmail.com (K.K.); adam.sieradzan.ug@gmail.com (A.S.); agnieszka_karczynska@wp.pl (A.K.); eiss84@gmail.com (M.M.-Z.); 2 SIB Swiss Institute of Bioinformatics, Quartier Sorge, Bâtiment Amphipole, CH-1015 Lausanne, Switzerland; Justyna.Iwaszkiewicz@isb-sib.ch (J.I.); Olivier.michielin@unil.ch (O.M.); vincent.zoete@sib.swiss (V.Z.); 3 Urology Research Unit, Urology Department, University Hospital of Lausanne (CHUV), CH-1011 Lausanne, Switzerland; Valerie.Cesson@chuv.ch; 4 Intercollegiate Faculty of Biotechnology UG&MUG, University of Gdansk, Abrahama 58, 80–308 Gdańsk, Poland; katarzyna.wegrzyn@biotech.ug.edu.pl; 5 Institute of Biochemistry and Biophysics, Polish Academy of Sciences, Pawińskiego 5a, 02–106 Warszawa, Poland; igor@ibb.waw.pl; 6 NanoBioMedical Center, Adam Mickiewicz University, Umultowska 85, 61–614 Poznań, Poland; 7 Department of Oncology, University Hospital of Lausanne (CHUV), Ludwig Cancer Research—Lausanne Branch, CH-1011 Lausanne, Switzerland; 8 Department of Oncology, University of Lausanne, Ch. des Boveresses 155, CH-1066 Lausanne, Switzerland; d.e.speiser@gmail.com; 9 Department of Fundamental Oncology, Lausanne University, Ludwig Institute for Cancer Research, Route de la Corniche 9A, CH-1066 Epalinges, Switzerland

**Keywords:** B-and T-lymphocyte attenuator, herpes virus entry mediator, immunotherapy, immune checkpoint inhibitor, disulfide-linked peptide, NMR structure, molecular docking, surface plasmon resonance

## Abstract

Immune checkpoints are crucial in the maintenance of antitumor immune responses. The activation or blockade of immune checkpoints is dependent on the interactions between receptors and ligands; such interactions can provide inhibitory or stimulatory signals, including the enhancement or suppression of T-cell proliferation, differentiation, and/or cytokine secretion. B-and T-lymphocyte attenuator (BTLA) is a lymphoid-specific cell surface receptor which is present on T-cells and interacts with herpes virus entry mediator (HVEM), which is present on tumor cells. The binding of HVEM to BTLA triggers an inhibitory signal which attenuates the immune response. This feature is interesting for studying the molecular interactions between HVEM and BTLA, as they may be targeted for novel immunotherapies. This work was based on the crystal structure of the BTLA/HVEM complex showing that BTLA binds the N-terminal cysteine-rich domain of HVEM. We investigated the amino acid sequence of HVEM and used molecular modeling methods to develop inhibitors of the BTLA/HVEM interaction. We synthesized novel compounds and determined their ability to interact with the BTLA protein and inhibit the formation of the BTLA/HVEM complex. Our results suggest that the HVEM (14–39) peptide is a potent inhibitor of the formation of the BTLA/HVEM protein complex.

## 1. Introduction

The recent development of immune checkpoint blockade therapies, which target regulatory pathways in T-cells to enhance antitumor immune responses, has led to important clinical advances and has provided a new weapon against cancer. The currently applied immunotherapies are mainly focused on blocking programmed cell death 1/programmed cell death-ligand 1 (PD1/PD-L1) [1,2,3] and cytotoxic T-lymphocyte antigen-4/cluster of differentiation 80/86 (CTLA-4/CD80/CD86) [4,5] interactions, but other immune checkpoints and their ligands could also be targeted. One of these is the B-and T-lymphocyte attenuator (BTLA) and its ligand, namely, the herpes virus entry mediator (HVEM). The binding of BTLA to HVEM decreases the activation of CD8^+^ T-cells [6,7,8]. The interaction of BTLA and HVEM negatively regulates immune responses, specifically T-cell responses. This has been suggested in different types of cancer, including gastric cancer [9], bladder cancer [10], hepatocellular carcinoma [11], and melanoma [12].

BTLA, a member of the immunoglobulin-like superfamily (IgSF), is a transmembrane glycoprotein composed of an extracellular domain [13], which binds with HVEM [7]. It is structurally and functionally similar to CTLA-4 and PD-1 [14]. The cytoplasmic domain of BTLA contains two immunoreceptor tyrosine-based inhibitory motifs (ITIM). The first intracellular event after the binding of BTLA to HVEM is the recruitment of tyrosine phosphatases (SHP-1 and 2) to bind with the ITIM domains, ultimately leading to the attenuation of effector functions [15].

HVEM belongs to the tumor necrosis factor receptor (TNFR) superfamily and is a transmembrane glycoprotein composed of four cysteine-rich domains (CRDs), each with about 40 amino acid residues. Three intrachain disulfide bridges, created by six highly conserved cysteine residues, stabilize the first three CRDs in HVEM. The fourth CRD contains only two disulfide bonds [16,17,18,19]. HVEM interacts not only with BTLA but also with cluster of differentiation 160 (CD160) [20,21], homologous to lymphotoxins, exhibits inducible expression, and competes with HSV glycoprotein D for herpes virus entry mediator, a receptor expressed by T lymphocytes (LIGHT), and lymphotoxin α (LTα) [22]. Similar to BTLA, binding of HVEM to CD160 triggers a blockade of T-cell activation, whereas interaction of HVEM with LTα and LIGHT leads to costimulation. Several studies have suggested that LIGHT and LTα interact with CRD2 and CRD3 of the HVEM protein, which is characteristic of TNFRs and their corresponding ligands [22]. In contrast, BTLA and CD160 bind with the CRD1 region of HVEM [20,23]. A recent study has shown that the inhibitory activity of HVEM through BTLA and CD160 seems to be dominant over the stimulatory activity of LIGHT [24].

A previous study demonstrated the crystal structure of the BTLA/HVEM complex (PDB code: 2AW2) [23]. It revealed key interactions between the proteins and identified “hot spots.” According to that study, BTLA binds with HVEM on two fragments: BTLA (amino acids 35–43) and BTLA (amino acids 118–128), which interact with the CRD1 domain of HVEM. The fragment HVEM (26–38) was shown to be directly involved in the binding of BTLA, and the N-terminal part of CRD1 does not participate in the formation of the protein complex [20]. The crystal structure of BTLA/HVEM shows that the interaction between both proteins is primarily stabilized by hydrogen bonds of the main chain and that there are few side-chain interactions [23].

In earlier years, researchers focused on the screening of small molecules as inhibitors of protein–protein interactions (PPIs) [1,25,26,27]. More recently, peptides have shown great potential as alternatives for inhibiting PPIs [28]. As described herein, we have designed and synthesized peptide inhibitors of the BTLA/HVEM complex formation, based on the HVEM binding fragment. We have also confirmed the ability of the synthesized peptide to interact with BTLA and inhibit the formation of the BTLA/HVEM complex, with the help of in silico and in vitro methods.

## 2. Results

### 2.1. Peptide Design

**Figure 1:**
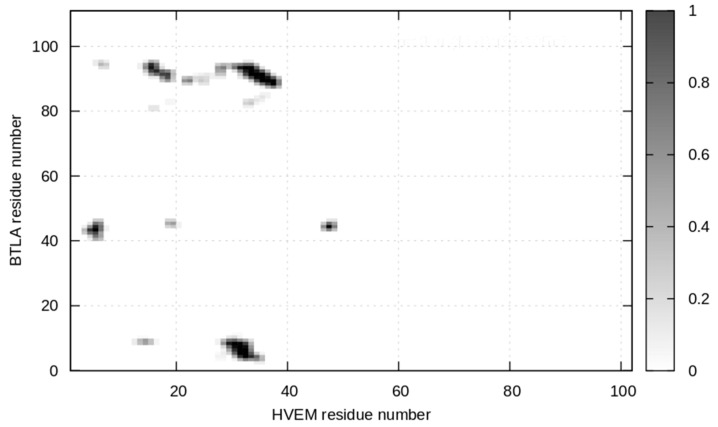
The contact map obtained for the interface between BTLA and HVEM from the crystal structure (PDB code: 2AW2). The gray scale uses black to indicate the highest contact value and white to indicate the smallest contact value.

To estimate the individual contribution of each residue from HVEM to the binding between BTLA and HVEM, we analyzed the contact map (colored in grayscale) between the two proteins based on the crystal complex (PDB code: 2AW2) (Figure 1) [23]. According to the results, 15 amino acids from HVEM are involved in interaction with BTLA. The following amino acid residues were found to have the highest number of contacts at the interface of the BTLA/HVEM complex formation: Glu6, Asp7, Glu8, Glu14, Pro17, Tyr23, Gly30, Glu31, Leu32, Thr33, Gly34, Thr35, Val36, Cys37, and Glu38. Based on this information, the HVEM (14–39) fragment was chosen for further studies as a potential inhibitor of BTLA/HVEM interactions.

### 2.2. Docking and Kinetic Studies for the HVEM Protein and HVEM (14–39) Peptide Based on the Crystal Structure of BTLA/HVEM Complex

**Figure 2:**
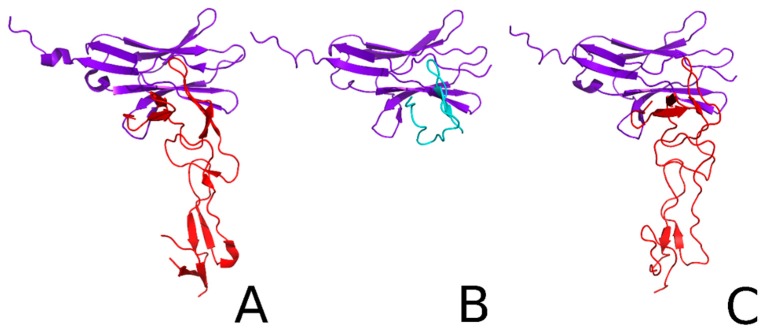
(**A**) Crystal structure of the B-and T-lymphocyte attenuator /herpes virus entry mediator complex (PDB code: 2AW2), in which the purple color indicates BTLA and red indicates HVEM. (**B**) The dominant cluster of the HVEM (14–39) peptide–BTLA complex obtained from UNRES simulation, in which the purple color indicates BTLA and cyan blue indicates the peptide. (**C**) The fourth cluster of the BTLA/HVEM complex obtained from UNRES simulation, in which HVEM and BTLA are highlighted as red and purple, respectively.

Using molecular docking with the UNited RESidue (UNRES) program, we checked whether the HVEM (14–39) peptide binds with BTLA in a similar way to the native HVEM protein (Figure 2). The results of the clustering reveal that the native-like cluster probability is 7.9% (fourth cluster) and 39.5% (first cluster) for HVEM and the HVEM (14–39) peptide, respectively. This indicates that the UNRES force field can predict the correct interface, but in the case of native HVEM, structures with the correct interface are sparse. The average root-mean-square deviation (RMSD) of those clusters is 1.68 Å and 1.41 Å for HVEM protein and the HVEM (14–39) peptide, respectively (Figure 2). Such accuracy is typically obtained by nuclear magnetic resonance (NMR) spectroscopy. Thus, molecular docking showed that the HVEM (14–39) peptide makes contacts with the BTLA protein which are similar to those of the native HVEM protein.

We then determined the kinetic constants (association and dissociation constants k_1_ and k_−1_, respectively) of the HVEM protein or HVEM (14–39) peptide binding to BTLA using UNRES canonical molecular dynamics (MD). The concentration of free HVEM or peptide, as a function of time, from these canonical simulations, is shown in Figure S1. From the fitted function, we determined k_1_ and k_−1_ for HVEM as equal to 16.895 and 0.0191, respectively (as the real-time is distorted in UNRES, these constants are left unitless). This leads to an equilibrium dissociation constant (K_D_) of 1.1 × 10^−3^. For the peptide, the association constant is 3 times smaller than that of the protein (k_1_ = 5.594); however, the dissociation rate constant is 19 times smaller than that of the protein (k_−1_ = 0.001). These data indicate that the HVEM (14–39) peptide binds slowly to BTLA (3 times more slowly than the HVEM protein does), but its interaction is almost 6 times stronger (K_D_ = 1.87 × 10^−4^) than that of the native HVEM protein (K_D_ = 1.1 × 10^−3^) and it may, therefore, be a potential inhibitor of the BTLA/HVEM complex formation.

### 2.3. Conformational Studies of HVEM (14–39) Peptide Using NMR Techniques

**Figure 3:**
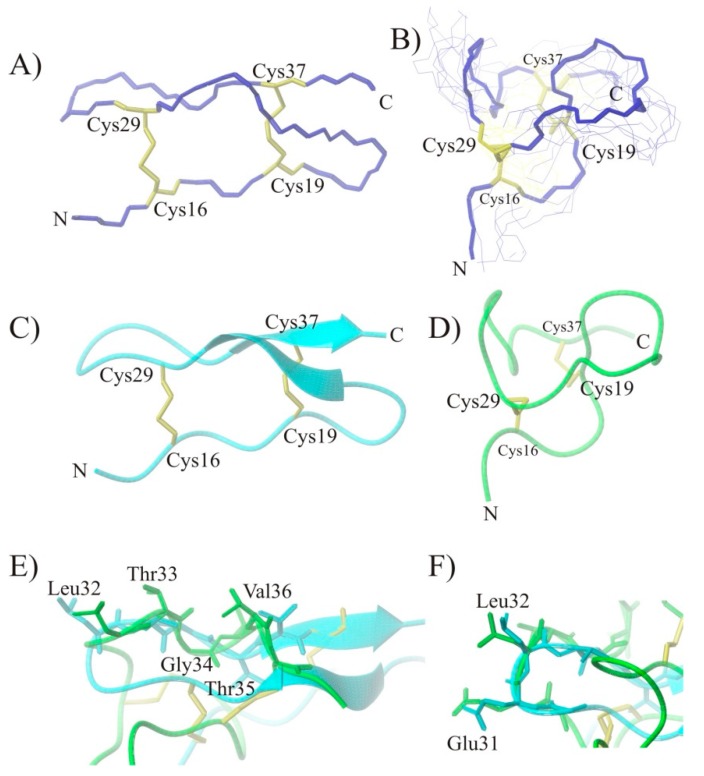
Panel A shows the spatial structure of the herpes virus entry mediator protein fragment (14–39) (PDB code: 2AW2). Panel B shows superimposed NMR conformations of all αC atoms of the HVEM (14–39) peptide (RMSD = 1.947 Å). The last structure from the MD has been emboldened. Panel C shows the structure of fragment 14–39 from the HVEM protein (PDB code: 2AW2) (cyan color), and panel D shows the NMR structure of the HVEM (14–39) peptide (green color) after MD calculations. Panel E shows the structure of fragment 32–36 from the HVEM protein (PDB code: 2AW2) (cyan color) with the NMR structure of the HVEM (14–39) peptide (green color) superimposed. The side chains of amino acid residues important for interaction with the BTLA protein are highlighted in the figure. Panel F shows the amino acid residues forming the bend. The structures are shown as a backbone (**A**,**B**), ribbon (**C**–**F**), or stick (**E**,**F**) representation; the cysteine side chains are shown in the stick representation. Disulfide bonds are shown in yellow.

The HVEM (14–39) peptide contains four cysteine residues (the cysteine residue in position 15 is replaced by serine) and could form inter- and intramolecular disulfide bonds during the synthesis. To obtain the compound with a position of disulfides, similar to the HVEM protein, orthogonal protecting groups on the cysteines were used. The NMR spectra where then registered and elucidated to confirm the structure of the peptide. The position of disulfide bridges was confirmed by the presence of nuclear Overhauser effects (NOEs) between the connected cysteine residues in the nuclear Overhauser effect spectroscopy (NOESY) (Figure S2). For the HVEM (14–39) peptide, NOE crosspeaks were observed between Cys16Hβ–Cys29Hβ (C16–C29 disulfide bond), between Leu32NH–Ser15Hα, and between Cys37NH–Cys19Hα and Cys37NH–Cys19Hβ (C19–C37 disulfide bond). For the studied peptide, it was possible to observe two sets of NMR chemical shifts for some residues, which is shown on the fingerprint regions of the NOESY spectra (Figure S3). This means that the HVEM (14–39) peptide contains flexible fragments. In the NMR spectra of the HVEM (14–39) peptide, the NOE crosspeaks in the region Cys16–Arg24 were strongly overlapped and therefore were not included in the structural analysis. Furthermore, in the C-terminus, the medium-range connectivities d_αN(*i,i+2*)_ and d_αN(*i,i+4*)_ indicate the formation of a turn in the Gly30–Thr35 region. The structural data from NMR were used to conduct MD simulations to determine the three-dimensional structure of the peptide. The NMR structure of HVEM (14-39) peptide contains a sequence of bend structures. It can be seen that the peptide, despite having two disulfide bridges, is flexible (Figure 3B). Significant mobility of the peptide is also confirmed by two sets of signals in the NMR spectra. However, it is interesting to note that the HVEM (14–39) peptide has the same topology as the fragment excised from the HVEM protein (i.e., −1, +2). Due to the greater freedom of the peptide, compared to that of the protein, it forms a wider structure (Figure 3B,D). In the NMR structure of the peptide, it is also seen that the distance between S–S bridges became shorter, whereby the connecting fragments formed a large loop in region 22–26 and a β-turn in region 30–33. These loops are exposed to the solvent of the opposite side of the structure of peptide. The loop in region 22–26 is stabilized by a series of electrostatic interactions and hydrogen bonds. In contrast, the β-turn in region 30–33 is stabilized by the hydrogen bond between the carbonyl oxygen atom of the Gly30 residue and the amide hydrogen of the Thr33 residue. Comparison of the NMR structure of the protein with that of the structure of the protein fragment indicates large similarities between the two structures in the region which is relevant for interaction with the BTLA protein (Figure 3E), where the side chains of amino acids are arranged in a similar way. The NMR structure of the HVEM (14–39) peptide also shows similarity to the structure of this fragment in the protein in the β-turn region 30–33 (Figure 3F). The presence of a bend structure in this region is confirmed by NMR spectra (NOE signals).

### 2.4. Docking and Determination of the Kinetic Constants of HVEM (14–39) Peptide Binding to BTLA Based on the NMR Structure of the Peptide

**Figure 4:**
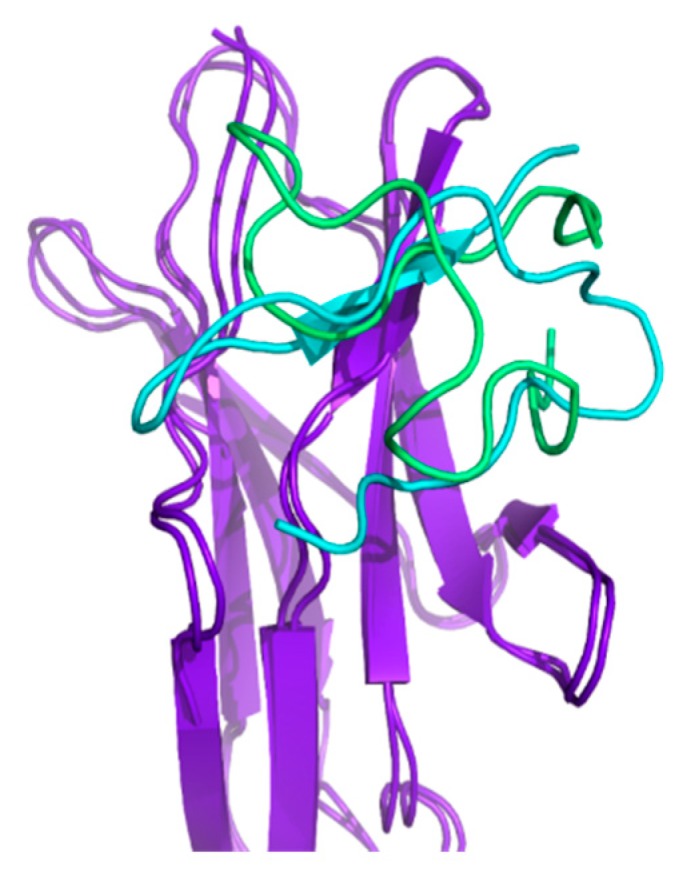
The comparison between the structure of the dominant cluster of HVEM (14–39) peptide docking to BTLA (purple) obtained from (i) simulations with peptide restraints based on the crystal structure (cyan) of the BTLA/HVEM complex and (ii) simulations with peptide restraints based on the structure derived from the nuclear magnetic resonance (NMR) spectra (green).

The results from multiplexed-replica exchange molecular dynamics (MREMD) simulations reveal that the NMR-based structure of HVEM peptide binds strongly to BTLA when compared with the crystal-based structure of peptide. The three dominant clusters have similar RMSD values of 5.07 Å, 5.09 Å, and 5.10 Å, respectively. The three dominant clusters constitute 68% of all conformations. Figure 4 shows a comparison between the dominant cluster obtained from HVEM (14–39) peptide simulations with (i) restraints of the peptide based on the crystal fragment of HVEM and (ii) peptide restraints based on the NMR structure.

As can be seen in Figure 4, the structure of both complexes is very similar. The binding pocket of BTLA is almost the same; however, the orientation of the peptide is dependent on the restraint. The kinetic analysis reveals that, in the case of the NMR-based structure, the peptide binds faster (k_1_ = 68.49) than the crystal-based structure (k_1_ = 5.594). The K_D_ values suggest also that NMR-based structure of peptide interacts with BTLA stronger than the crystal-based structure of peptide (K_D_ = 1.69 × 10^−4^ for the NMR-based structure; K_D_ = 1.87 × 10^−4^ for the crystal-based structure). Figure S1 shows a comparison of the concentration of the unbound peptide against time for different types of restraints.

### 2.5. Experimental Studies of the Interactions Between the HVEM (14–39) Peptide and BTLA Protein

**Figure 5:**
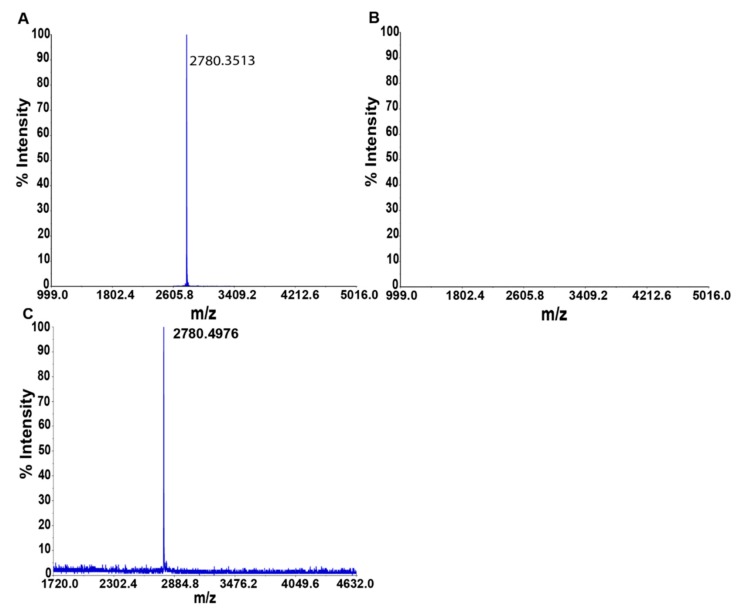
Affinity test results for the HVEM (14–39) peptide in a microcolumn with immobilized BTLA-Fc protein. The figure shows: (**A**) supernatant; (**B**) last wash; and (**C**) elution.

The ability of the HVEM (14–39) peptide to interact with BTLA was tested using affinity chromatography and surface plasmon resonance (SPR). In the affinity test, the peptide was incubated in a microcolumn containing immobilized BTLA protein. Excess peptide was then removed, and dissociation of the complex was performed. Three fractions were analyzed using mass spectrometry: supernatant, last wash, and elution. In the supernatant (Figure 5A) and elution fraction (Figure 5C), the signals m/z from the peptide were present, whereas in the last wash there was no signal detected (Figure 5B), suggesting that the HVEM (14–39) peptide can interact with the BTLA protein.

**Figure 6:**
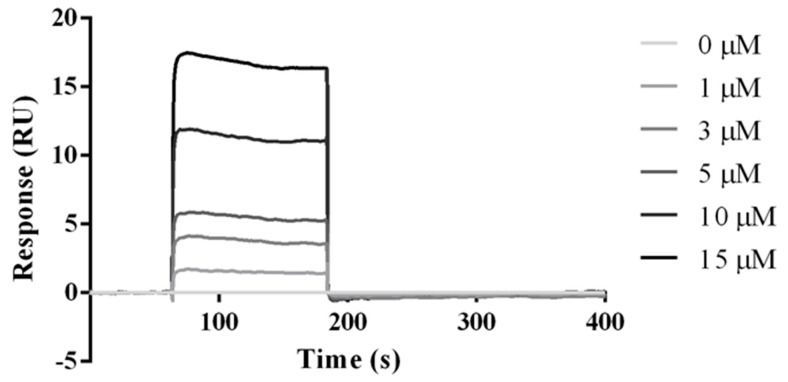
SPR analysis of the BTLA protein binding to the HVEM (14–39) peptide. Sensorgrams show the results of SPR analysis conducted on the binding of the indicated concentrations of BTLA protein (C = 0, 1, 3, 5, 10, and 15 μM) in HBS-EP buffer. Constants were determined based on four measurements: k_1_ = 7.64 × 10^4^ 1/Ms ± 6.81 × 10^4^; k_−1_ = 3.32 × 10^−3^ 1/s ± 1.55 × 10^−3^; K_D_ = 1.02 × 10^−7^ M ± 0.97 × 10^−7^, where k_1_ is the association rate constant, k_−1_ is the dissociation rate constant, and K_D_ = k_1_/k_−1_.

To analyze the binding properties between BTLA and the HVEM (14–39) peptide, we performed the affinity measurements using SPR. Thus, the peptide extended by five glycine residues at the N-terminus and biotin was first immobilized on a streptavidin matrix-coated sensor chip. The BTLA protein was then injected at different concentrations (0, 1, 3, 5, 10, and 15 µM). Figure 6 shows the selected equilibrium binding curve for the binding of BTLA to the HVEM (14–39) peptide (all data are presented in Table S1). Our results indicate that BTLA binds directly to the HVEM (14–39) peptide with K_D_ = 102 × 10^−9^ (102 nM), which is 2.45 times stronger than that of native HVEM protein (K_D_ = 250 × 10^−9^, 250 nM) [29]. Moreover, the SPR results reveal that the peptide binds approximately 8 times slower (k_1_ = 76400), and dissociates 36 times slower (k_−1_ = 0.00332) than does the HVEM protein (k_1_ = 620000 and k_−1_ = 0.12), which agrees with the theoretical results of simulations with crystal-based structure and NMR-based structure of the peptide (Table 1). The SPR results suggest that the HVEM (14–39) peptide is a potent inhibitor of the BTLA/HVEM complex formation.

### 2.6. Studies of the Inhibitory Properties of the HVEM (14–39) Peptide in a Cell Line Assay

**Figure 7:**
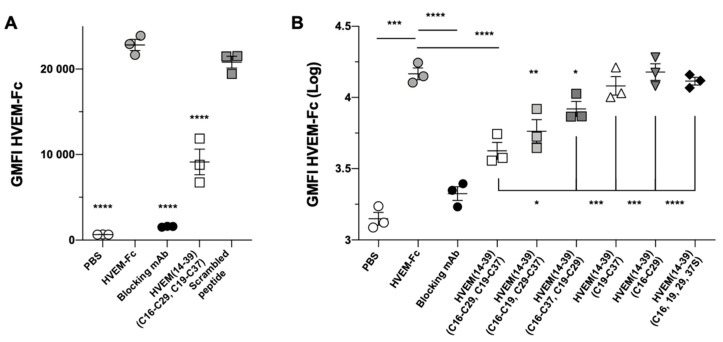
HVEM (14–39) peptide blocks BTLA/HVEM binding. 293T cells expressing human BTLA were incubated with peptides (5 mg/mL) prior to labeling with HVEM-Fc and AF647-conjugated anti-human IgG antibody. The blocking capacity of the HVEM (14–39) peptide was first compared to (**A**) a scrambled (Ac-SECGRCEAPEKTKSLCVTPEPVGCYG-NH_2_) peptide or (**B**) to variant peptides (Table S2). The graph shows the geometric fluorescence intensity (GMFI). * *p* < 0.05, ** *p* < 0.01, *** *p* < 0.001 and **** *p* < 0.0001 following one-way ANOVA and Dunn’s post-test, compared to the HVEM-Fc condition in A and HVEM-Fc or native peptide in B.

Finally, we determined the ability of the HVEM (14–39) peptide to inhibit the formation of the BTLA/HVEM complex at the cellular level, using 293T cells expressing the human BTLA protein [30]. Prior to the analysis, we confirmed the stability of the peptide in culture medium and PBS at 4 and 37 °C (Figures S4 and S5). Titration assay showed that the best inhibitory properties were observed at 5 mg/mL (Figure S6). We observed that the HVEM (14–39) peptide significantly blocks the binding of HVEM to BTLA but not the scrambled peptide (Figure 7A).

We then assessed the impact of the disulfide bridges (C16–C29 and C19–C37) of the peptides on their blocking capacity. To do this, we synthesized and tested various peptides with different disulfide bridge positions: with two disulfide bridges, namely, HVEM (14–39)^C16–C19^, ^C29–C37^ and HVEM (14–39)^C16–C37^, ^C19–C29^, with only one disulfide bridge, namely, HVEM (14–39)^C19–C37^ and HVEM (14–39)^C16–C29^, and without any disulfide bridge, namely, HVEM (14–39)^C16,19,29,37S^ (the sequences of the peptides are given in Table S2). Figure 7B shows that there was a slight decrease in the blocking capacity when the disulfide bridges were altered. Moreover, the blocking capacity was completely lost when only one or no disulfide bridge was present in the peptide. This clearly highlights the key role of the disulfide bridges in the capacity of the native HVEM (14–39) peptide to block the BTLA/HVEM interaction.

Although 5 mg/mL is a strong concentration, we did not observe any toxicity of the peptide on 293T cells (data not shown). To ascertain this result, we cultured peripheral blood mononuclear cells (PBMC) from healthy individuals with or without the HVEM (14–39) peptide for 6 and 24 h. Then, we measured the cell death by counting the cells using trypan blue and by flow cytometry using 4′,6-diamidino-2-phenylindole dihydrochloride (DAPI). We did not find any significant increase in cell death (Figure S7), suggesting that the HVEM (14–39) peptide is not toxic for immune cells, even at high concentration.

## 3. Discussion

Blocking immune checkpoints using monoclonal antibodies has revolutionized cancer immunotherapy. Several new compounds, such as antibodies and small molecules (including peptides and peptidomimetics) targeting PD-1 or CTLA-4 or its ligands, have been described in the literature [25,31]. There are many more studies conducting clinical trials [32]. In this study, we focused on other inhibitory receptor–ligands: BTLA and HVEM. To date, there is no literature on peptides/peptidomimetics that are able to effectively block BTLA/HVEM interactions. Based on in silico and in vitro methods, we have shown that the HVEM (14–39) peptide can efficiently block ligation between BTLA and HVEM.

It has been shown that the binding site of the HVEM protein interacting with BTLA is located in the CRD1 domain, which is composed of about 40 amino acids and is stabilized by three intermolecular disulfide bridges [20,23]. The third and fourth beta strands of HVEM are directly involved in the protein interaction. The binding fragment of HVEM has two cysteine residues at positions 29 and 37, which in the native protein form disulfide bridges with cysteine residues at positions 16 and 19, respectively. The third disulfide bond is formed between the amino acids at positions 4 and 15 and stabilizes the N-terminal part of HVEM, which does not participate in BTLA/HVEM interactions. However, it stabilizes the tertiary structure of the protein. The most important residues of HVEM are Pro17, Tyr23, and Val36 while residues of medium importance are Glu8, Lys26, and Glu31 [23]. Cheung and colleagues confirmed the importance of Lys26 and additionally pointed out the importance of Arg24 and Glu27 [33]. In a previous report, we observed that the HVEM (23–39) fragment was able to block BTLA/HVEM interaction in enzyme-linked immunosorbent assays (ELISA) but not in cellular assays. This blocking capacity was due to free Cys present in the peptide [30]. Therefore, we selected the HVEM (14–39) fragment encompassing seven of the critical residues for further analyses and identified the two disulfide bridges at positions Cys16–Cys29 and Cys19–Cys37. In addition, Cys15 was replaced by serine in order to decrease the binding effect due to free Cys.

By using molecular modeling (the UNRES force shield method), we showed that the HVEM (14–39) peptide (crystal-based structure) binds to BTLA in the same position as the HVEM protein and interacts with BTLA 5.88 times more strongly than does the HVEM protein (Table 1; K_D_ BTLA/HVEM crystal-based structure (No. 3) and K_D_ BTLA/HVEM (14–39) crystal-based structure (No. 4) = 5.88). This suggests that the peptide may be a potent inhibitor of the BTLA/HVEM complex formation. The NMR data indicate that the conformation of the peptide is not the same as in the protein, despite the fact that it possesses two disulfide bridges. However, there is a similarity in the arrangement of the peptide fragment (Leu32–Cys37) directly interacting with BTLA, which takes on a stretched structure. In addition, the structure of the peptide obtained from NMR was docked to the BTLA protein, and the data indicate that it interacts with BTLA in the same place as the HVEM protein (Figure S8) and even stronger than was calculated for the peptide crystal-based structure (Table 1; K_D_ BTLA/HVEM (14–39) crystal-based structure (No. 4) and K_D_ BTLA/HVEM (14–39) NMR-based structure ((No. 5) = 1.12) [23].

The interaction between the HVEM (14–39) peptide and BTLA was then confirmed using an affinity test with immobilized BTLA protein. SPR provided more accurate data. The value of K_D_ determined for the HVEM (14–39) peptide immobilized on streptavidin sensor and BTLA was 102 nM. According to the literature, the value of K_D_ measured for immobilized HVEM and BTLA proteins was 250 nM (HVEM amino acid sequences from L1 to Y103) (Table 1) [29]. K_D_ and affinity are inversely related, which means that the lower the K_D_ value, the higher the affinity of the molecules. The HVEM (14–39) peptide binds and dissociates from BTLA significantly more slowly than does the HVEM protein and interacts with BTLA 2.45 times more strongly than does HVEM (Table 1; K_D_ BTLA/HVEM (No. 1) and K_D_ BTLA/HVEM (14–39) (No. 2) = 2.45). The fact that the HVEM (14–39) peptide binds more slowly, dissociates more slowly, and binds more strongly to BTLA is revealed by both theoretical and SPR experimental methods. The stronger binding of the HVEM (14–39) peptide to BTLA compared to the binding of the HVEM protein to BTLA could be due to the larger flexibility of the peptide, which probably translated into a stronger adaptability to the binding sites of BTLA.

The inhibitory properties of the peptide were also tested using functional cellular assays. The HVEM (14–39) peptide inhibited the BTLA/HVEM complex formation in a dose-dependent manner (Figure S6). The results suggest that the HVEM (14–39) peptide is a competitive inhibitor and competes with the HVEM protein for binding with BTLA. Moreover, analogs of the HVEM peptide with rearranged disulfide bridges or without such bridges showed reduced blocking properties. Notably, the loss of one of the two disulfide bridges (peptides HVEM (14–39)^C16–C29^ and HVEM (14–39)^C19–C37^) was enough to abrogate the blocking capacity. This result suggests that a single disulfide bond may not be enough to stabilize the tertiary structure of the peptide.

Overall, we have shown that the HVEM (14–39) peptide is an inhibitor of BTLA/HVEM binding. However, further studies are needed to determine its blocking capacity in an immunological context.

## 4. Materials and Methods

### 4.1. Recombinant Molecules and Antibodies

This study was approved by the ethics committee of the canton of Vaud, Switzerland (protocol #2019-00546; 14/03/2019).

Recombinant human BTLA protein was purchased from Novoprotein (company product code: C563). Recombinant human BTLA-Fc, HVEM-Fc and anti-human BTLA (#7.1)-blocking mAb were a generous gift from Prof. D. Olive (Inserm U1068/CNRS U7258, Marseille, France) [34]. Anti-HVEM mAb was biotinylated according to the manufacturer’s instructions (Biotin Labeling Kit-NH_2_, Abnova, #KA0003).

### 4.2. Docking of the HVEM Protein or HVEM (14–39) Peptide to BTLA

The HVEM (14–39) peptide structure was assumed to as in the crystal structure as the peptide contains two disulfide bonds significantly rigidifying the structure. Docking of the HVEM protein or the HVEM (14–39) peptide to BTLA was performed with an UNRES force field [35,36] by using MREMD with weak restraints on the torsional and valence angles of the backbone and weak restraints on the side chain–side chain and peptide group–peptide group contacts, based on the native structure of the monomers presented in the protocol [37]. It should be noted that no restraints were imposed on the protein–protein interface. There were 20 starting structures, each consisting of two molecules randomly oriented with respect to each other. The temperatures 250, 260, 270, 280, 285, 290, 295, 300, 305, 310, 315, 320, 330, 340, 350, 360, 370, 380, 390, and 400 K, with two trajectories per temperature, were used. Each simulation comprised 10,000,000 steps with a time step of 0.49 fs, which corresponds to 5 ns of UNRES time, which in turn corresponds approximately to 5 µs of real-time [38,39]. Next, weighted histogram analysis method (WHAM) was performed as well as clustering at a temperature of 270 K to obtain 10 final structures of both complexes. The representative cluster was the structure closest to the ensemble average.

### 4.3. Determination of the Kinetic Constants of HVEM or HVEM (14–39) Peptide Binding to BTLA

To determine the association and dissociation rate constants, 100 canonical molecular dynamics simulations were performed with a Berendsen thermostat at 300K using an UNRES force field. Structures were then assigned to two states: a proper quaternary structure and an improper quaternary structure. The assignment was performed based on the RMSD of the structure. The cutoff was based on the results of clustering corresponding to the sum of the average RMSD of the most native-like cluster and the maximum distance of the within-cluster family: these were 5.24 Å and 4.41 Å for HVEM and the HVEM (14–39) peptide, respectively.

Next, an equation for second-order kinetics (Equation) was fitted to the concentration of free HVEM or the free HVEM (14–39) peptide (or improperly bound to BTLA) as a function of time. The derivation of the equation is shown in Table S3.

(1) $$\left[E\right]=\frac{{\left[E\right]}_{0}\left(\frac{k_{-1}}{2k_{1}}+\;\sqrt{k}+\left(-\frac{k_{-1}}{2k_{1}}+\sqrt{k}\right)exp\left(-2k_{1}t\sqrt{k}\right)\right)}{{\left[E\right]}_{0}+\frac{k_{-1}}{2k_{1}}+\sqrt{k}-\left({\left[E\right]}_{0}+\frac{k_{-1}}{2k_{1}}-\sqrt{k}\right)exp\left(-2k_{1}t\sqrt{k}\right)}$$

(2) $$k=\frac{k_{-1}^{2}+4k_{1}k_{-1}{\left[E\right]}_{0}}{4k_{1}^{2}}$$

where [*E*] is the concentration of unbound (or improperly bound) HVEM or the HVEM (14–39) peptide, [*E*]_0_ is the initial concentration of HVEM, *k*_1_ is the association constant, and *k*_−1_ is the dissociation constant.

### 4.4. Peptide Synthesis

All peptides were synthesized on a semiautomated peptide synthesizer (Millipore 9050 Plus PepSynthesizer, Millipore Corporation, Burlington, VT, USA) using the method of solid-phase peptide synthesis (SPSS). Peptides were synthesized on a TentaGel R RAM resin (0.19 mmol/g) using Fmoc/tBu chemistry, standard amino acid derivatives, and a combination of protected cysteine residues (Fmoc-Cys(Acm)-OH and Fmoc-Cys(Trt)-OH). Acetylation of the N-terminal amino group of the peptide was performed using 1-acetylimidazole (1.10 g/1 g of resin at room temperature for 24 h). The peptide was cleaved from the resin for 2 h using a mixture of 88% TFA, 5% phenol, 5% deionized water, and 2% triisopropylsilane (10 mL/1 g of resin at room temperature for 2 h). After filtration of the exhausted resin, the solution was concentrated under vacuum, and the residue was triturated with Et_2_O. The precipitated peptide was centrifuged for 15 min at 4000 rpm, followed by decantation of the ether phase from the crude peptide (this process was repeated thrice). The peptide was then dissolved in H_2_O and lyophilized.

### 4.5. Peptide Purification

Prior to the purification process, the peptides were dissolved in water and a 10-fold excess of dithiothreitol (DTT) was added. The mixture was kept in an ultrasonic bath at 60 °C for 30 min. The crude peptide was purified by reverse-phase high-performance liquid chromatography (RP-HPLC) on a semi-preparative Phenomenex Luna C8 (2) (250 mm × 20 mm, 5 μm) column. The aqueous system (A) consisted of 0.1% (*v/v*) TFA in water, and the organic phase (B) consisted of 80% acetonitrile in water containing 0.08% (*v/v*) TFA. A linear gradient from 5% B to 50% B in A for 120 min was applied. Purification was monitored by UV absorption at wavelengths of 222 nm and 254 nm. The purity of the peptide was verified by liquid chromatography coupled with electrospray ionization, ion trap, and time-of-flight mass spectroscopy (LC ESI–IT–TOF MS, Shimadzu, Shimpol, Warsaw, Poland) and RP-HPLC with a Kromasil C8 analytical column (250 mm × 4.6 mm, 5 μm), using a gradient of 5% to 100% B in A for 60 min (A and B as described above).

### 4.6. Formation of Disulfide Bonds

First, the disulfide bonds were formed between cysteine residues protected by trityl groups (removed during the cleavage conditions). After purification, the peptide was dissolved in a mixture of H_2_O and methanol (1:9, *v*:*v*), at a concentration of 40 mg/L. The pH was adjusted and maintained between 8 and 9 using ammonia while stirring the solution at room temperature for 7 days in the presence of atmospheric oxygen. The solvents were then evaporated and the peptides were lyophilized. Reaction progress was verified using analytical RP-HPLC and LC ESI–IT–TOF MS (conditions: see peptide purification). Next, the second disulfide bonds were formed, and the peptides were dissolved in a solution of acetic acid, methanol, and H_2_O (1:9:1, *v*:*v*:*v*) at a concentration of 40 mg/L. Iodine (25 to 50 fold excess) was dissolved in methanol and was added to the solution, which was then stirred at room temperature for 7 days. The mixture was filtered by using a Dowex ion exchange resin, and the excess iodine was removed. The progress of the reaction was monitored by analytical RP-HPLC and LC ESI-IT–TOF MS (conditions: see peptide purification). The solvents were then evaporated and the peptides were lyophilized. The peptides were repurified using RP-HPLC under conditions previously described.

### 4.7. Nuclear Magnetic Resonance Spectroscopy and NMR Structure Calculation

HVEM (14–39) samples were prepared by dissolving 6 mg of peptide in 0.5 mL of phosphate buffer (pH 7.4). All experiments were performed on an Agilent DDR2 800 MHz spectrometer operated at 18.8 T (^1^H resonance frequency 799.94 MHz). The spectrometer was equipped with four channels, a Performa IV *z*-gradient block, a ^1^H/^13^C/^15^N probe head with inverse detection and a temperature unit. Due to the low dispersion of resonances, the NMR experiments were conducted within the temperature range of 20–35 °C for every 5 °C. The data collected contained two-dimensional homonuclear TOCSY and NOESY experiments recorded with 80 ms and 120 ms, respectively. The ^13^C chemical shifts were evaluated from ^1^H−^13^C heteronuclear single quantum coherence (HSQC) spectra collected with a natural abundance of ^13^C isotopes. Structural analysis of the peptide was performed on experimental data acquired at 25 °C, which revealed a slightly better dispersion of signals. All ^1^H and ^13^C chemical shifts were referenced in an indirect manner with respect to the external standard sodium 2,2-dimethyl-2-silapentane-5-sulfonate (DSS) using 0.251449530 ratios for ^13^C resonances [40]. The recorded NMR data were processed by NMRPipe software [41] and analyzed with the SPARKY program [42]. The assignments of ^1^H and ^13^C resonances were performed by a standard approach based on joint analysis of TOCSY, NOESY, and ^1^H-^13^C HSQC spectra [43]. Inspection of NOESY spectrum yielded 88 (38 intra-residue, 33 sequential, 6 medium-range, and 11 long-range) distance constraints. The initial structures were calculated with the CYANA (version 3.98) program [44] provided to 20 structures out of 200 calculated chosen on the base lowest target function. In the next stage, the MD simulations of the final structure obtained from the CYANA program were conducted with the AMBER force field in the AMBER 14.0 package [45] using a protocol including time-averaged NMR restraints (TAV) derived from NMR spectroscopy with the force constant for interproton connectivity equal to 50 kcal/(mol × Å^2^). No torsion angle restraints were used in the calculations. The geometry of the peptide groups was kept all trans with a force constant of f = 50 kcal/mol × rad^2^, according to the NMR data. The coordinates were collected every 2000 steps. The structures obtained in the last 0.5 ns of the MD simulation were considered for further analysis. All the figures were evaluated using the MOLMOL program [46]. NMR data have been deposited at the BioMagResBank (number 28031).

### 4.8. Docking and Determination of the Kinetic Constants of the HVEM (14–39) Peptide to BTLA Based on the NMR Structure of the Peptide

After obtaining the experimental structure of the HVEM (14–39) peptide, determination of docking and kinetic constants was repeated as described earlier with the use of restraints based on the structure determined by NMR. The assignment of bound structures was performed based on the RMSD of the structure. The cutoff was based on the results of the clustering corresponding to the sum of the average RMSD of the most native-like cluster and the maximum distance of the within-cluster family, which equals 6.67 Å.

### 4.9. Preparation of Microcolumn and Affinity Test

Immobilization of the BTLA protein in a microcolumn and the affinity test were performed according to the procedure described in our previous studies [30]. The mass spectroscopy (MS) measurements were conducted using a MALDI-TOF/TOF 5800 (AB Sciex, Germany) instrument. α-Cyano-4-hydroxycinnamic acid (CHCA) was used as the matrix (10 mg/mL, Sigma-Aldrich, Saint Louis, MO, USA). The measurements were conducted in reflector positive mass mode with previous mass calibration with a commercial standard peptide mixture (The Peptide Mass Standards Kit for Calibration of AB SCIEX MALDI-TOF™ Instruments).

### 4.10. SPR Analysis

Standard SPR analyses were performed using a Biacore T200 (GE Healthcare) instrument essentially as described in the manufacturer’s manual. BTLA protein binding was studied using the HVEM(14–39) peptide elongated by five glycine residues at the N-terminus and immobilized on a streptavidin matrix-coated Sensor Chip SA (GE Healthcare). The peptide was immobilized on the sensor surface to yield a final value of ~1100 RU. HBS-EP (150 mM NaCl, 10 mM HEPES pH 7.4, 3 mM EDTA, 0.05% Surfactant P20) was used as the running buffer. In all the experiments, the buffer flow was set to 30 µL/min, with all injections at a volume of 120 µL. The sensorchip surface was regenerated with 0.5 M NaCl and 10 mM glycine. The data obtained were analyzed using Biacore T200 Evaluation Software 3.1 (Biacore, GE Healthcare, USA). The results are presented as sensorgrams obtained after subtraction of the background response signal from control experiments with buffer injections.

### 4.11. BTLA/HVEM Interaction—Cell Line Assay

Cells (293T) expressing BTLA (a generous gift from Dr. C. Krummenacher, University of Pennsylvania, USA) were maintained in culture with RPMI 10% FCS supplemented with 0.2 mg/mL hygromycin B. The cells (2 × 10^5^) were incubated with HVEM-Fc (at 8 μg/mL in PBS containing 0.5% BSA) for 30 min at 4 °C. The cells were then washed and incubated with goat anti-human Fc conjugated with AF647 for 20 min at 4 °C in PBS containing 0.5% BSA (Jackson ImmunoResearch, #109–606–170). Inhibition of BTLA/HVEM binding by different peptides was assessed after preincubating the cells with different concentrations of the peptide (5, 1, and 0.1 mg/mL) for 1 h at 4 °C in PBS. After washing in PBS, the cells were stained for 20 min at 4 °C with an amine reactive dye (aqua live/dead stain kit, from Life Technologies, L34957) for dead cell exclusion. BTLA expression was controlled by anti-BTLA APC-conjugated antibody (Biolegend, #344510). Maximum inhibition of binding was determined by adding anti-BTLA7.1 antibody (2 μg/mL) for 1 h at 4 °C, before adding HVEM-Fc. Sample acquisition was performed on a Gallios Flow Cytometer (Beckman Coulter), and data were analyzed using FlowJo Software (TreeStar).

### 4.12. Stability of the HVEM (14–39) Peptide

Stability in PBS: The HVEM (14–39) peptide was incubated in PBS at concentrations of 0.1, 1, and 5 mg/mL. The incubation time points were 0, 2, 3, 6, and 24 h at 4 °C and 37 °C. Then, samples were analyzed by RP-HPLC on a Kromasil C8 analytical column (250 mm, 4.6 mm, 5 μL) using a linear gradient from 5% B to 100% B in A in 60 min (A and B: see purification of peptides). All stability tests were performed at least in triplicate.

Stability in the medium: 50 µL of the HVEM (14–39) peptide (concentrations 0.1, 1, and 5 mg/mL) was added to 200 µL of medium (RPMI 10% FCS). The incubation time points were 0, 2, 3, 6, and 24 h at 4 and 37 °C. The samples were precipitated by the addition of 4-fold excess of absolute ethanol (v/v), incubated on ice for 15 min and centrifuged at 18,000 rpm for 20 min at 4 °C. After centrifugation, the supernatant was collected and dried under vacuum. Then, samples were resuspended in 120 µL of 0.1% TFA and analyzed by RP-HPLC under the same conditions as described above. All stability tests were performed at least in triplicate.

### 4.13. Cytotoxic Assay

Blood samples were also collected from healthy volunteers through the local Swiss blood bank. Fresh peripheral blood mononuclear cells were purified by density gradient centrifugation (Isopaque-Ficoll) within 2 h after blood sampling. Cells were cryopreserved in RPMI medium 1640 supplemented with 40% FCS and 10% DMSO.

Then, 2 × 10^4^ total PBMC per well (96-well plate) was cultured with or without the HVEM (14–39) peptide at 5 or 0.1 mg/mL in RPMI 1640 supplemented with 10% FCS, 1% penicillin-streptomycin, and 25 mM HEPES. After 6 and 24 h at 37 °C, cells were harvested counted with Trypan blue exclusion. Cells were then labeled with DAPI and analyzed by flow cytometry to determine the percentage of cell death.

## Figures and Tables

**Table 1:** Rate constants: k_1_, k_−1_ and K_D_ for the BTLA/HVEM and BTLA/HVEM (14–39) complexes determined using SPR and molecular dynamics simulation. The comparison of K_D_ BTLA/HVEM (No. 1) and K_D_ BTLA/HVEM (14–39) (No. 2) = 2.45; K_D_ BTLA/HVEM crystal-based structure (No. 3) and K_D_ BTLA/HVEM (14–39) crystal-based structure (No. 4) = 5.88; K_D_ BTLA/HVEM crystal-based structure (No. 3) and K_D_ BTLA/HVEM (14–39) NMR-based structure (No. 5) = 6.51; K_D_ BTLA/HVEM (14–39) crystal-based structure (No. 4) and K_D_ BTLA/HVEM (14–39) NMR-based structure (No. 5) = 1.12. The theoretical k_1_ i k_−1_ were determined with use of equation 1 and 2.

No.	System	Method	k_1_	k_−1_	K_D_
1	BTLA/HVEM	SPR	620,000	0.12	2.5 × 10^−7^
2	BTLA/HVEM (14–39)	SPR	76,400	0.00332	1.02 × 10^−7^
3	BTLA/HVEM _crystal-based structure_	Molecular dynamics	16.895	0.0190	1.1 × 10^−3^
4	BTLA/HVEM (14–39) _crystal-based structure_	Molecular dynamics	5.594	0.00105	1.87 × 10^−4^
5	BTLA/HVEM (14–39) _NMR-based structure_	Molecular dynamics	68.49	0.0116	1.69 × 10^−4^

## Supplementary Materials

The following are available online at https://www.mdpi.com/1422-0067/21/2/636/s1.

## Abbreviations

BTLA	B-and T-lymphocyte attenuator
CHCA	α-cyano-4-hydroxycinnamic acid
CD160	cluster of differentiation 160
CRD	cysteine-rich domain
CSI	chemical shift index
CTLA-4	cytotoxic T-lymphocyte antigen-4
DAPI	4′,6-diamidino-2-phenylindole dihydrochloride
DSS	sodium 2,2-dimethyl-2-silapentane-5-sulfonate
DTT	dithiothreitol
ELISA	enzyme-linked immunosorbent assay
Fmoc/tBu	9-fluorenylmethoxycarbonyl/tert-butyl
HSQC	heteronuclear single quantum coherence
HVEM	herpes virus entry mediator
IgSF	immunoglobulin-like superfamily
ITIM	immunoreceptor tyrosine-based inhibitory motif
LC ESI–IT–TOF MS	liquid chromatography coupled with electrospray ionization, ion trap, and time-of-flight mass spectroscopy
LIGHT	homologous to lymphotoxins, exhibits inducible expression, and competes with HSV glycoprotein D for herpes virus entry mediator, a receptor expressed by T lymphocytes
LTα	lymphotoxin α
mAb	monoclonal antibody
MS	mass spectroscopy
MALDI-TOF	matrix-assisted laser desorption ionization, time-of-flight
MREMD	multiplexed-replica exchange molecular dynamics
MD	molecular dynamics
NOE	nuclear Overhauser effect
NOESY	nuclear Overhauser effect spectroscopy
NMR	nuclear magnetic resonance
PBS	phosphate-buffered saline
PD-1	programmed cell death 1
PDB	protein data bank
PD-L1	programmed cell death-ligand 1
PBMC	peripheral blood mononuclear cells
PPIs	protein–protein interactions
RP-HPLC	reverse-phase high-performance liquid chromatography
RMSD	root-mean-square deviation
SPPS	solid-phase peptide synthesis
SHP-1 and 2	Src homology-2-containing protein 1 and 2
SPR	surface plasmon resonance
TFA	trifluoroacetic acid
TNFR	tumor necrosis factor receptor
TOCSY	total correlation spectroscopy
UNRES	UNited RESidue
WHAM	weighted histogram analysis method
