# Supplementary material for: Disulfide-Linked Peptides for Blocking BTLA/HVEM Binding
Source: Int J Mol Sci. 2020 Jan 18;21(2):636. doi: 10.3390/ijms21020636 (PMC7013932; doi:10.3390/ijms21020636)
Supplement: Supplementary file 1 [file ijms-21-00636-s001.pdf]

Supplemental Data

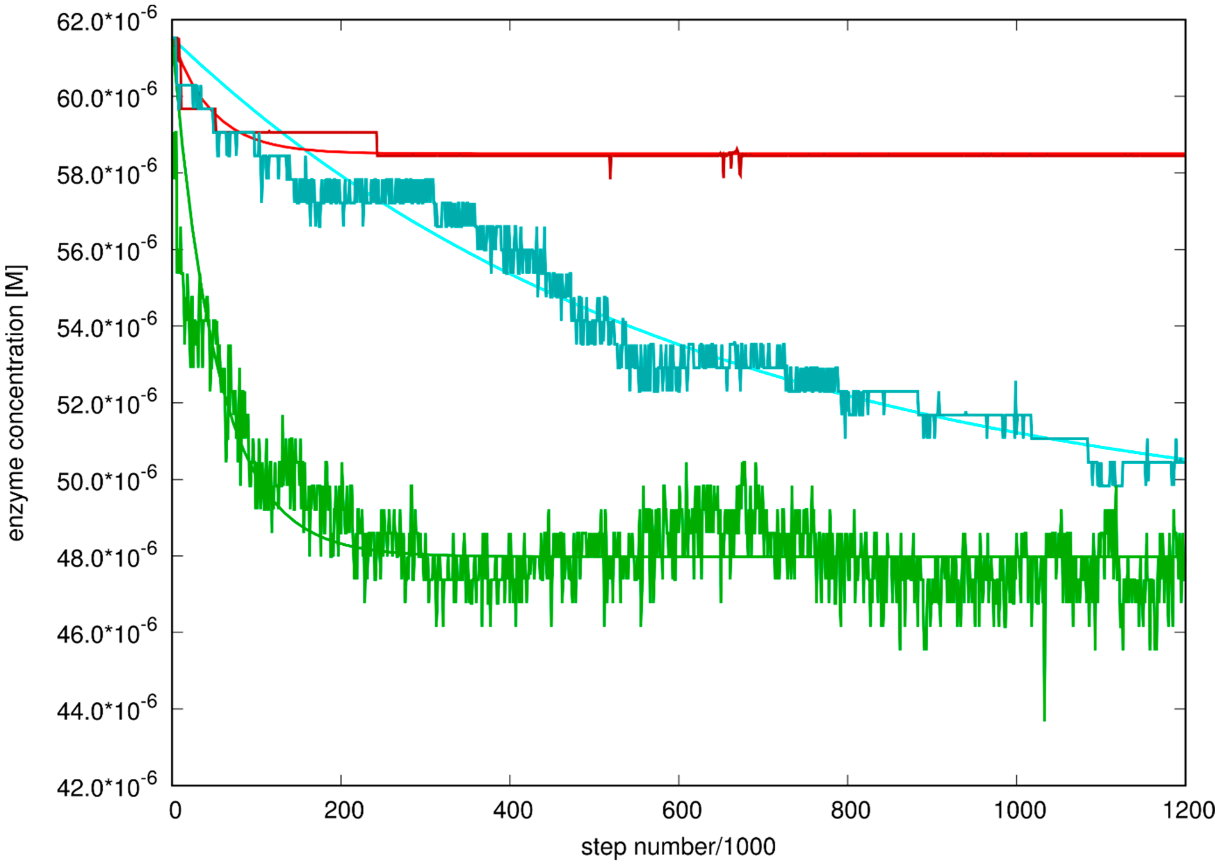

Fig. S1. Concentration of unbound structures against over time plot for HVEM (dark red line) and peptide (light blue line) with restrains based on crystal structure of BTLA/HVEM complex and peptide (green) simulated with restrains based on the NMR structure; second order equation corresponding fitted curves to obtained data for HVEM (red) and for peptide with restrains based on crystal structure of BTLA/HVEM complex (cyan) and for peptide with restrains based on NMR structure of this peptide (green).

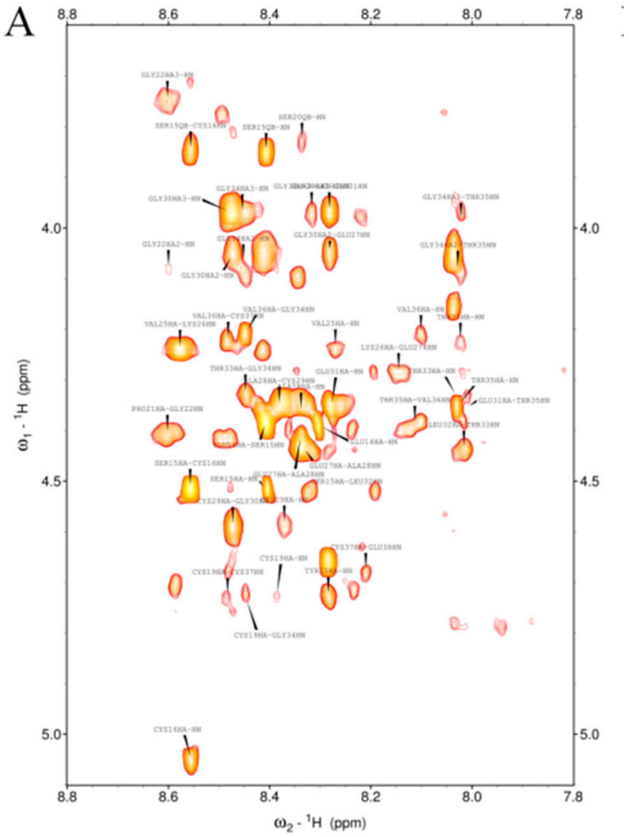

Fig. S2. The HN-Hα region of NOESY spectra (range 7.7 to 8.8 ppm) with the sequential assignments of all residues and numbered crosspeaks of the HVEM(14-39) peptide in phosphate buffer at 25°C.

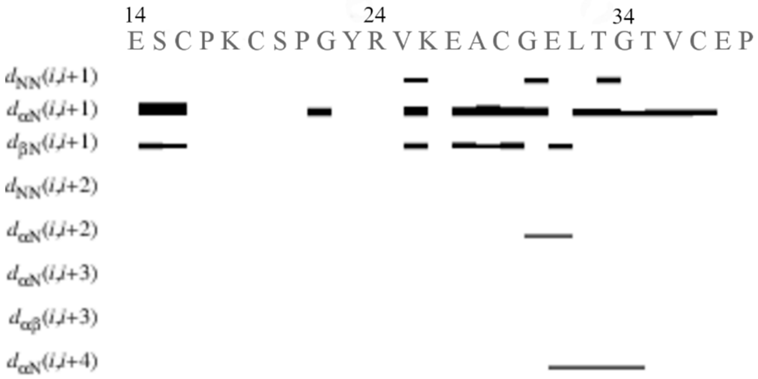

Fig. S3. The NOE effects corresponding to interproton distances (the height of the bar is proportional to the signal volume) in phosphate buffer at 25°C for the HVEM(14-39) peptide.

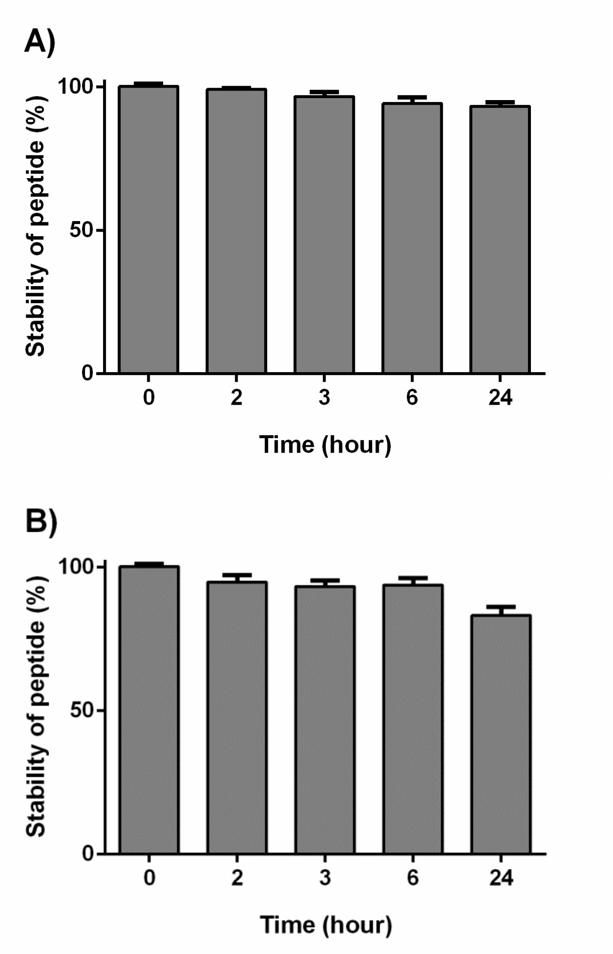

Figure S4. The peptide stability (mean ± SEM) in A) PBS buffer, B) medium was determined using RP-HPLC. The analysis was done by comparing the area under peaks in a control sample (peptide dissolved in water, time=0) and a sample after incubation in PBS or medium at time points: 0 h, 2 h, 3 h, 6 h, 24 h at 4 and 37°C. The experiments were performed for the three different peptide concentrations: 0.1, 1 and 5 mg/ml. For all concentrations of HVEM(14-39) peptide at 4° and 37°C the results were very similar (here the data for concentration 1 mg/ml at 4°C are presented).

A)

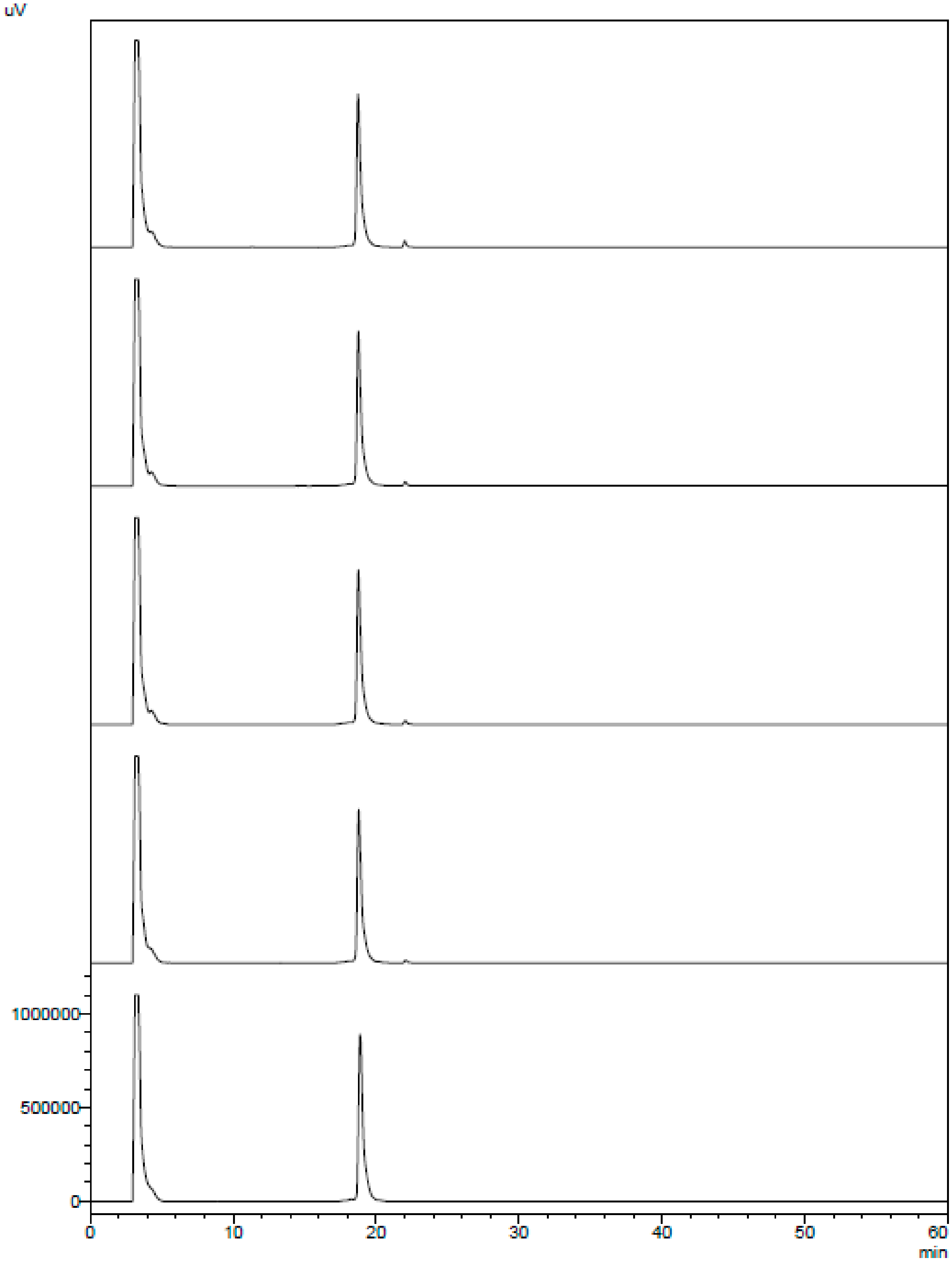

B)

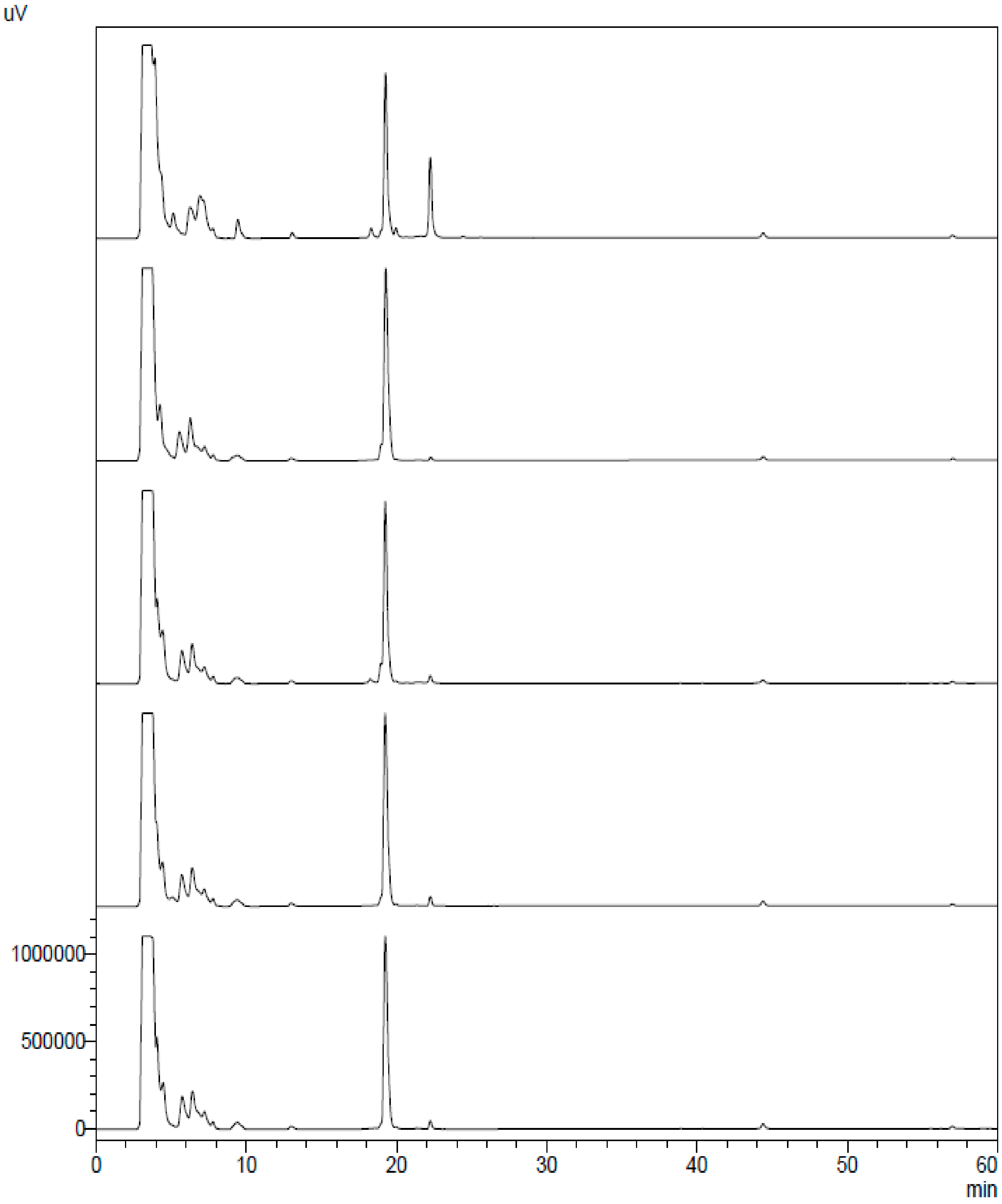

Figure S5. Chromatograms registered for HVEM(14-39) peptide incubated in A) PBS, B)

medium at time points (from the bottom) = 0h, 2h, 3h, 6h and 24h (gradient 5-100%B in 60 min).

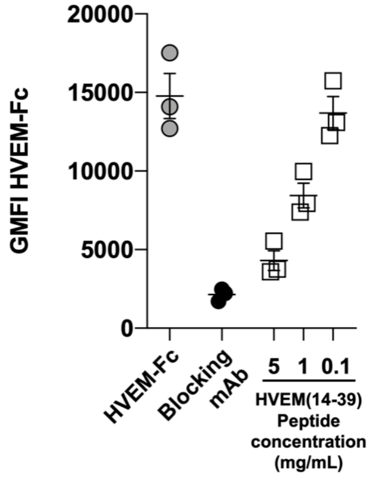

Figure S6. 293T cells expressing human BTLA were incubated with different concentrations (0.1, 1 and 5 mg/ml) of peptide HVEM(14-39) prior labeling with rhHVEM-Fc and AF647-conjugated anti-human IgG antibody.

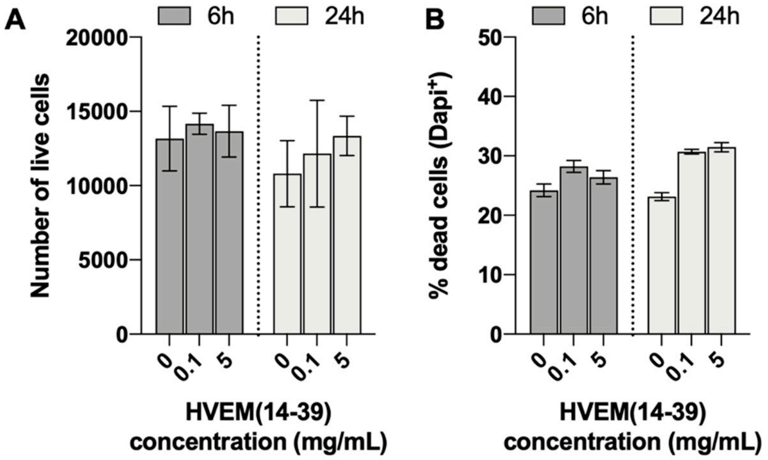

Fig. S7. Cytotoxic assay. PBMC from 3 healthy donors were incubated with HVEM(14-39) peptide at 0, 0.1 and 5 mg/ml in culture medium for 6 and 24h. Cells were subsequently counted and labelled with a dead cell marker. Statistical analysis: one-way ANOVA (Kruskal-Wallis) followed by Dunn’s test to compare each concentration to the control (0 mg/mL).

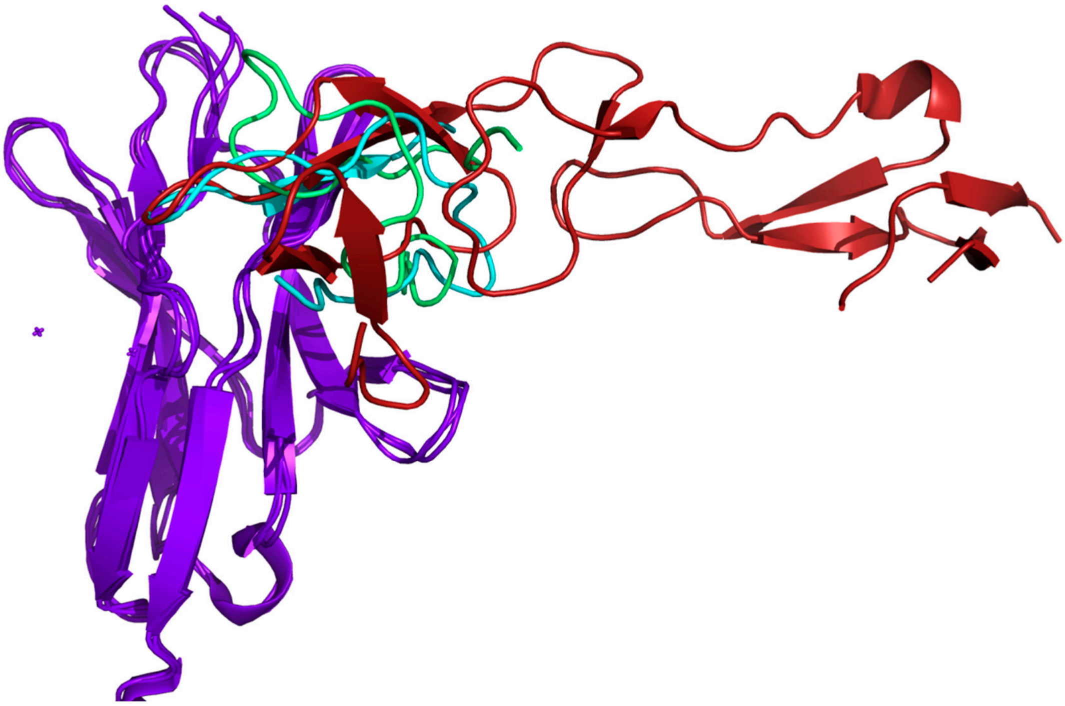

Fig. S8. Superimposed structures of complexes of BTLA protein (purple) with peptides (cyan and green) and with HVEM (red). Complex structures were superimposed after the structure of the BTLA protein.

Table S1. Kinetics parameters for the interactions between HVEM(14-39) peptide and BTLA protein.

No	k_1 (1/Ms)	k_{-1} (1/s)	K_D (M)
**1.**	1,06E+05	1,37E-03	1,30E-08
**2.**	2,84E+04	4,23E-03	1,49E-07
**3.**	1,31E+04	2,83E-03	2,17E-07
**4.**	1,59E+05	4,85E-03	3,06E-08
**Average**	**7,64E+04**	**3,32E-03**	**1,02E-07**
**StdDev**	6,81E+04	1,55E-03	9,74E-08

Table S2. The amino acid sequences of the peptides.

PEPTIDE	AMINO ACID SEQUENCE
HVEM(14-39)	Ac-ESCPKCSPGYRVKEACGELTGTVCEP-NH_2
HVEM(14-39)$^{C16\text{-}C19,\ C29\text{-}C37}$	Ac-ESCPKCSPGYRVKEACGELTGTVCEP-NH_2
HVEM(14-39)$^{C16\text{-}C37,C19\text{-}C29}$	Ac-ESCPKCSPGYRVKEACGELTGTVCEP-NH_2
HVEM(14-39)$^{C16\text{-}C29}$	Ac-ESCPKCSPGYRVKEACGELTGTVCEP-NH_2
HVEM(14-39)$^{C19\text{-}C37}$	Ac-ESCPKCSPGYRVKEACGELTGTVCEP-NH_2
HVEM(14-39)^{C16,19,29,37S}	Ac-ESSPKSSPGYRVKEASGELTGTVSEP-NH_2
HVEM(14-39)^{SCR}	Ac-SECGRCEAPEKTKSLCVTPEPVGCYG-NH_2

Table S3. Derviation of associtation and dissociation rate constant for complex formation reaction.

Rates for the reactions:

$$\mathrm{I + E} \underset{k_{-1}}{\overset{k_1}{\rightleftarrows}} \mathrm{E \cdot I}$$

are

$$\frac{dE}{dt} = \frac{dE}{dt} = -k_1[E][I] + k_{-1}[EI] \quad (1)$$

$$\frac{dEI}{dt} = -k_{-1}[EI] + k_1[E][I] \quad (2)$$

as in our case concentration of enzyme is equal to concentation of inhibitor ([E]=[I]) the equation can be simplified to:

$$\frac{dE}{dt} = \frac{dE}{dt} = -k_1[E]^2 + k_{-1}[EI] \quad (3)$$

$$\frac{dEI}{dt} = -k_{-1}[EI] + k_1[E]^2 \quad (4)$$

Moreover:

$$[E] + [EI] = [E]_0 + [EI]_0 \quad (5)$$

and as $[EI]_0 = 0$ therefore

$$[E] + [EI] = [E]_0$$

$$[EI] = [E]_0 - [E] \quad (6)$$

By substitution 6 to equation 3 we obtain:

$$\frac{dE}{dt} = -k_1[E]^2 + k_{-1}[E]_0 - k_{-1}[E] \quad (7)$$

By separation of variable we obtain:

$$\frac{dE}{-k_1[E]^2 + k_{-1}[E]_0 - k_{-1}[E]} = dt \quad (8)$$

Which is typical integral of $\frac{1}{ax^2+bx+c}$ where a is $-k_1$ b is $-k_{-1}$ and c is $k_{-1}[E]_0$ and with subtitution of $u = [E] + \frac{k_{-1}}{2k_1}$ and $du = dx$ and $k = \frac{k_{-1}^2 + 4k_1k_{-1}[E]_0}{4k_1^2}$ we can write 8 integer in a simplified form:

$$\frac{1}{k_1} \int \frac{1}{u^2 - k} = t \quad (9)$$

As k_1 and k_{-1} are positive values, after integration we obtain:

$$\frac{1}{-2k_1\sqrt{k}} \ln \frac{u - \sqrt{k}}{u + \sqrt{k}} + C = t \quad (10)$$

After returning with u to original values we get:

$$\frac{1}{-2k_1\sqrt{k}}\ln\frac{[E]+\frac{k_{-1}}{2k_1}-\sqrt{k}}{[E]+\frac{k_{-1}}{2k_1}+\sqrt{k}}+C=t \tag{11}$$

To determine C we know that at time $t=0$, $[E]=[E]_0$, therefore C is:

$$\frac{1}{2k_1\sqrt{k}}\ln\frac{[E]_0+\frac{k_{-1}}{2k_1}-\sqrt{k}}{[E]_0+\frac{k_{-1}}{2k_1}+\sqrt{k}}=C \tag{12}$$

Therfore:

$$\frac{1}{-2k_1\sqrt{k}}\ln\frac{[E]+\frac{k_{-1}}{2k_1}-\sqrt{k}}{[E]+\frac{k_{-1}}{2k_1}+\sqrt{k}}+\frac{1}{2k_1\sqrt{k}}\ln\frac{[E]_0+\frac{k_{-1}}{2k_1}-\sqrt{k}}{[E]_0+\frac{k_{-1}}{2k_1}+\sqrt{k}}=t \tag{13}$$

after transformation we obtain:

$$\exp(-2k_1t\sqrt{k})=\frac{([E]+\frac{k_{-1}}{2k_1}-\sqrt{k})([E]_0+\frac{k_{-1}}{2k_1}+\sqrt{k})}{([E]+\frac{k_{-1}}{2k_1}+\sqrt{k})([E]_0+\frac{k_{-1}}{2k_1}-\sqrt{k})}=\frac{W}{Z} \tag{14}$$

where W is

$$\begin{aligned} W &= [E][E]_0+[E]\frac{k-1}{2k_1}+[E]\sqrt{k}+\frac{k^2-1}{4k_1^2}+[E]_0\frac{k-1}{2k_1}-[E]_0\sqrt{k}-k \\ &= [E][E]_0+[E]\frac{k-1}{2k_1}+[E]\sqrt{k}-[E]_0\sqrt{k}-[E]_0\frac{k-1}{2k_1} \end{aligned} \tag{15}$$

and Z is

$$\begin{aligned} W &= [E][E]_0+[E]\frac{k-1}{2k_1}-[E]\sqrt{k}+\frac{k^2-1}{4k_1^2}+[E]_0\frac{k-1}{2k_1}+[E]_0\sqrt{k}-k \\ &= [E][E]_0+[E]\frac{k-1}{2k_1}-[E]\sqrt{k}+[E]_0\sqrt{k}-[E]_0\frac{k-1}{2k_1} \end{aligned} \tag{16}$$

Therefore equation 14 can be writen as:

$$\begin{aligned} &[E]([E]_0+\frac{k_{-1}}{2k_1}-\sqrt{k})\exp(-2k_1t\sqrt{k})+[E]_0(\sqrt{k}-\frac{k_{-1}}{2k_1})\exp(-2k_1t\sqrt{k})= \\ &[E]([E]_0+\frac{k_{-1}}{2k_1}+\sqrt{k})-[E]_0(\sqrt{k}+\frac{k_{-1}}{2k_1}) \end{aligned} \tag{17}$$
